# Timing of Psychopharmacological and Nutritional Interventions in the Inpatient Treatment of Anorexia Nervosa: An Observational Study

**DOI:** 10.3390/brainsci11091242

**Published:** 2021-09-19

**Authors:** Jacopo Pruccoli, Martina Pelusi, Giorgia Romagnoli, Elisabetta Malaspina, Filomena Moscano, Antonia Parmeggiani

**Affiliations:** 1 IRCCS Istituto delle Scienze Neurologiche di Bologna, Centro Regionale per i Disturbi della Nutrizione e dell’Alimentazione in Età Evolutiva, U.O. di Neuropsichiatria dell’Età Pediatrica, 40138 Bologna, Italy; jacopo.pruccoli@studio.unibo.it (J.P.); martina.pelusi2@studio.unibo.it (M.P.); elisabetta.malaspina@aosp.bo.it (E.M.); filomena.moscano@aosp.bo.it (F.M.); 2 Dipartimento di Scienze Mediche e Chirurgiche (DIMEC), Università di Bologna, 40138 Bologna, Italy; giorgia.romagnoli2@studio.unibo.it

**Keywords:** antipsychotics, naso-gastric tube, anorexia nervosa, feeding and eating disorders, timing, childhood, adolescence

## Abstract

This study aims to investigate possible different outcomes in the inpatient treatment of anorexia nervosa (AN) related to different timings of psychopharmacological and nutritional interventions. A retrospective observational study was conducted, involving young patients hospitalized for AN, treated with naso-gastric tube feeding (NGT). Participants were divided into five groups according to early (0–7 days) or late (8+ days) introduction of atypical antipsychotics (AAP) and NGT: early AAP-early NGT (EE), early AAP-late NGT (EL), late AAP-early NGT (LE), late AAP-late NGT (LL) and a control group treated with NGT only (NGT). Concurrent clinical and treatment variables were analyzed. AN psychopathology was measured with the Eating Disorder Inventory-3 (EDI-3) EDRC score. Outcomes were assessed as admission-discharge body-mass index (BMI) improvement and length of hospital stay (LOS). Contributions of variables related to outcomes were assessed with multifactorial-analyses of variance (MANOVA). Seventy-nine patients were enrolled in the study. LOS was different among treatment groups (F (4, 75) = 5.993, *p* < 0.001), and EE patients showed lower LOS than LE (*p* < 0.001) and LL (*p* = 0.025) patients. BMI improvement was not significantly different among treatment groups but correlated negatively with age (F (1, 72) = 10.130, *p* = 0.002), and admission BMI (F (1, 72) = 14.681, *p* < 0.001). In conclusion, patients treated with early AAP and early NGT showed lower LOS than those treated with late AAP. Prognostic treatment variables should be investigated in wider samples.

## 1. Introduction

Anorexia nervosa (AN) is a mental health condition, which is characterized by food avoidance with significant weight loss and altered perception of one’s own body and most commonly begins during childhood and adolescence [1]. AN entails numerous psychiatric and medical comorbidities, such as anxiety disorders, mood disorders, personality disorders, autism spectrum disorder, and cardiovascular problems [2,3,4,5,6].

Even though inpatient treatment may promote weight restoration in AN regardless of the model of care provided, the most recent guidelines indicate the need for additional research to determine the critical treatment elements related to weight restoration [7]. Current guidelines mostly recommend nasogastric tube feeding (NGT) if a meal plan and supplemental nutrition drinks are not managed in malnourished patients [8,9,10,11]. Excessively rapid weight restoration in underweight patients has been linked to such metabolic urgencies as refeeding syndrome [12]. Literature lacks guidance on the appropriate or advisable timing for specific interventions, such as NGT feeding, geared toward weight restoration in patients with AN.

Atypical antipsychotics (AAP) are frequently prescribed medications for patients with AN. However, according to the National Institute for Clinical Excellence guidance for the recognition and treatment of eating disorders [13], antipsychotics and other medications should not be used as the sole treatment for anorexia nervosa. Recent guidelines suggest the use of some AAP in the management of low weight patients, but there is a paucity of data regarding the efficacy and tolerability of these agents [7]. Reasons behind the use of AAP include the delusional thinking in acute cases with AN, the neurobiological conceptualizations of psychotic and cognitive symptoms in eating disorders, and the results of clinical trials of antipsychotic medications in adult patients with AN [14]. AAP could assist in managing the anxiety and depressive symptoms of AN, and their known propensity to cause weight gain could be beneficial in underweight individuals [15]. A specific effect on physical hyperactivity has been documented as well [16]. AAP may have a role promoting weight recovery in severe patients, especially when applied in specific therapeutic contexts, e.g., hospitalization [17]. Proper timing for the use of antipsychotics has been repeatedly debated, mainly with respect to the treatment of schizophrenia [18]. Some researchers have discouraged early antipsychotic interventions, since prodromal psychotic symptoms may normalize independently of the administration of drugs [19,20]; antipsychotics may also provoke unwanted brain morphological modifications [21] and represent a risk for stigmatization [22]. Other studies have encouraged early treatments with AAP on account of their efficacy with relatively low side effects [23]. This also supports the notion of psychoses as developmental disorders [24] that can be modified by early interventions.

This study aims to investigate possible outcome differences among interventions with AAP and NGT administered with different timings, in a group of child and adolescents hospitalized for AN in an Italian third-level regional centre for the care of children and adolescents affected by eating disorders.

## 2. Materials and Methods

### 2.1. Methods

This is a retrospective study, which considers patients who referred to our Regional Centre for Feeding and Eating Disorders in children and adolescents in Bologna between 1 January 2016 and 31 December 2020. Inclusion criteria were (a) diagnosis of AN according to the Diagnostic and Statistical Manual of Mental Disorders—fifth edition (DSM-5) criteria [1]; (b) hospitalization with inpatient treatment regime; (c) treatments with AAP and with NGT feeding, both started during hospitalization; (d) acquisition of informed consent. Exclusion criteria were (a) treatment with NGT present at admission but started outside our centre, and (b) lack of a complete clinical documentation. Only treatments with NGT lasting 7 or more days were considered in the study.

**Figure 1:**
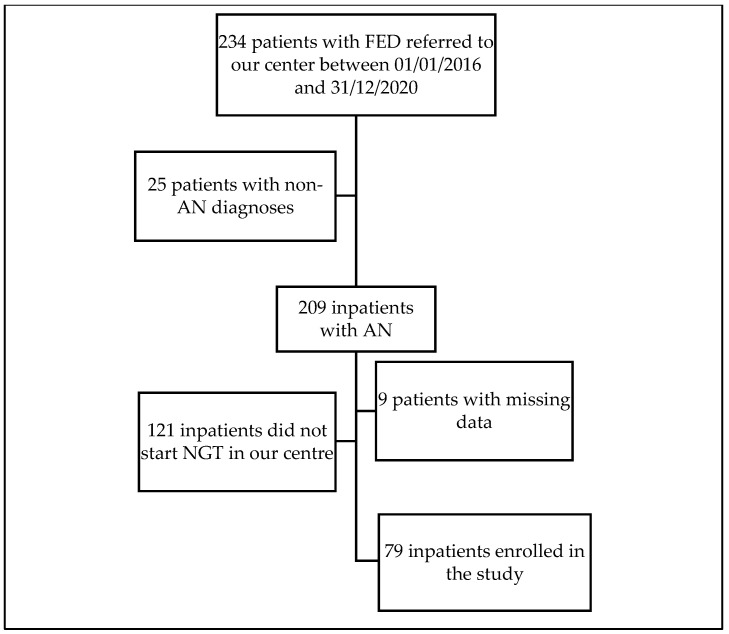
Flowchart of the study. Abbreviations: AN: anorexia nervosa; FED: feeding and eating disorders; NGT: naso-gastric tube; FED: feeding and eating disorders.

The clinical documentation of 234 patients with feeding and eating disorders referred to our centre between 1 January 2016 and 31 December 2020 was thoroughly reviewed. The flowchart of the study is reported in Figure 1. Among these patients, data required to assess inclusion and exclusion criteria (diagnosis, treatment regime, timing of AAP and NGT treatments) were retrospectively available for 225 (96.2%) of the eligible the patients. The remaining 9 patients (3.8%), mainly individuals hospitalized for emergency treatments and rapidly transferred to other departments, were discharged from the study. Seventy-nine eligible patients were enrolled in the study.

### 2.2. Measures

Diagnoses of AN were performed by clinicians trained in the field of FED. All the patients were subjected to a psychological, nutritional, and psychiatric inpatient treatment regime. Demographic and clinical data were obtained for all patients. Body mass index (BMI) at hospital admission and discharge was recorded. Duration of illness before hospitalization and duration of hospitalization were also considered. Psychopathology for eating disorders was assessed using the Eating Disorder Inventory-3 (EDI-3) [25], a test validated in the Italian language for the use in patients with eating disorders [26]. For the purpose of this study, we considered the composite score eating disorder risk (EDRC) at admission, resulting from the combination of the subscales drive for thinness, bulimia, and body dissatisfaction.

As for the treatment with AAP, we noted down the following items: types of AAP, length of treatment, maximum dosage, and major adverse-effect reactions (ADR). As for the treatment with NGT, we recorded the length of treatment. The patients were divided into groups according to their early (0–7 days) or late (>7 days) inpatient treatment starting time with AAP and NGT. Thus, five independent groups of patients were formed, namely: early AAP-early NGT (EE), early AAP-late NGT (EL), late AAP-early NGT (LE), late AAP-late NGT (LL) and a control group treated with NGT, but no AAP. The concurrent administration of selective serotonin reuptake inhibitors (SSRI) when inpatients’ treatment required it was recorded.

Given the observational and retrospective nature of this study, the choice to assign different patients to specific treatment groups was determined only by clinical reasons according to Italian and international guidelines [13,27]; no patient was deliberately selected or randomized to be assigned to specific treatment groups.

Difference in discharge-admission BMI and length of in-hospital stay (LOS) (expressed in days) represented the treatment outcomes.

The cut-off point between early and late treatment introduction was chosen according to two main literature and clinical reasons: (1) refeeding syndrome occurs more frequently during the first week of specialized nutritional treatment, with up to 27.5% of patients developing hypophosphatemia during this time interval [28,29,30]. Thus, treatment decisions concerning NGT during this cut-off entail relevant clinical implications; (2) one week (seven days) represents the time cut-off indicated by the DSM-5 when assessing the current severity of most psychotic disorders, such as brief psychotic disorder, schizophreniform disorder and schizophrenia [1], and this cut-off has been used to practically define transition to psychosis in prospective studies [31]. Given its relevance for patients with psychotic symptoms and its potential relevance for rapid AAP introduction, this cut-off was adopted in our study, since similar cut-offs have not been defined in the field of eating disorders.

### 2.3. Ethical Aspects

The study was approved by the local Ethical Committee (Comitato Etico di Area Vasta Emilia Centro della Regione Emilia-Romagna, CE-AVEC) (Protocol Code NPI-DAPSIFA 2020, study code 9/2021/Oss/AOUBo).

### 2.4. Statistical Analysis

Descriptive statistics for demographic and clinical variables included means and standard deviations, or absolute and percentage frequencies. Student *t*-tests were used for continuous variables when appropriate (Mann–Whitney test for non-parametric distributions); and the Chi-square test was used for categorical variables. Bonferroni correction for multiple comparisons was applied. The patients were divided into five groups according to the starting time of AAP and NGT treatment during hospitalization, and descriptive analyses for demographic and clinical variables were provided for each group. Shapiro–Wilk’s and Levene’s tests were used to assess normality of data distribution and homogeneity of variance. Comparisons of clinical variables among the groups were conducted using analyses of variance (ANOVA) with Bonferroni post-hoc analyses. Then, Pearson’s r or Spearman’s rho correlations were calculated between outcome measures (BMI difference, LOS) and clinical/treatment variables across the entire sample. The relationships and the relative weight of variables significantly associated with the outcome measures were then studied with multifactorial-ANOVA (MANOVA), followed by single analyses of covariance (ANCOVA). Post-hoc analyses were conducted adopting the Bonferroni post-hoc test. Welch’s correction and Games–Howell procedure were applied when homogeneity of variance assumption was violated. The significance level was set at 0.05, and all tests were two-tailed. All statistical analyses were conducted using JASP, version 0.14.1 for Windows.

## 3. Results

### 3.1. Demographic and Clinical Variables

Seventy-eight (98.7%) of the participants were females and one (1.3%) male. The mean age was 14.6 (±2.0) years. As for the frequencies of AN subtypes, 78 (98.7%) of the patients presented AN, restrictive subtype, while 1 patient (1.3%) presented AN, binge-purging subtype. The mean duration of untreated illness (DUI) was 14.8 (±11.5) months. A comorbid diagnosis was documented in 10 patients, namely obsessive-compulsive disorder (6 patients, 7.6%), major depressive disorder (3 patients, 3.8%) and panic disorder (1 patient, 1.3%). The mean EDI-3-EDRC score at admission was 72.9 (±20.2). The mean BMI at admission was 13.7 (±1.7) kg/m^2^, while the mean BMI at discharge was 15.6 (±1.7) kg/m^2^, with a mean discharge-admission difference of 1.9 (±1.4) kg/m^2^. Sixty-eight patients (86.0%) were treated with AAP, while 11 patients (14.0%) received NGT only and represented a control group. AAP were early introduced after admission (0–7 days) in 30 patients (44.1%), while a later introduction (>7 days) was adopted in 38 patients (55.9%). The mean number of days between admission and AAP initial administration was 25.6 (±30.9).

All the patients were treated with NGT. A treatment with NGT was early introduced after admission (0–7 days) in 43 patients (54.4%), and later (>7 days) in 36 patients (45.6%). The mean number of days between admission and the introduction of NGT was 16.7 (±27.5). The patients were divided into five groups. Demographic and clinical characteristics of the five groups of patients are reported in Table 1.

### 3.2. Psychopharmacological Treatments

Among the patients receiving AAP, the most frequent AAP used in the sample was olanzapine (27 patients, 39.7%). None of the patients received a treatment with two or more AAP concurrently. In 5 patients (7.4%) the treatment with an AAP was switched to a different AAP during hospitalization. As for antidepressant treatments, 75 patients (94.9%) were treated with an SSRI during hospitalization: 65 (82.3%) received sertraline, 6 patients (7.6%) were treated with fluvoxamine, and 18 patients (22.7%) received fluoxetine. Two patients (2.5%) experienced adverse drug reactions, namely somnolence (1 patient, treated with olanzapine and sertraline) and hypotension (1 patient, treated with olanzapine and sertraline).

### 3.3. Outcome Measures and Clinical Variables

Correlations between outcome variables and demographic, clinical, treatment and outcome variables are reported in Table 2. BMI difference was negatively correlated to age at admission and BMI at admission. No variable was correlated to LOS.

Differences in outcome among the five treatment groups are reported in Table 1. Differences in outcomes among AAP are reported in Table 3.

### 3.4. Differences in Outcome Measures among Treatment Groups

A significant difference emerged among treatment groups as regards treatment outcomes (LOS and BMI difference) (F(1, 75) = 3.941, *p* < 0.001).

#### 3.4.1. Admission-Discharge BMI Difference

Age and BMI at admission were significantly correlated with BMI difference. Thus, the comparison of BMI difference among treatment groups was conducted controlling for these two potential confounding factors. The ANCOVA on BMI difference revealed no significant difference among treatment groups (F (4, 72) = 1.879, *p* = 0.123). Instead, the BMI difference was significantly correlated with age (F (1, 72) = 10.130, *p* = 0.002), and with BMI at hospitalization (F (1, 72) = 14.681, *p* < 0.001). Full comparisons of treatment outcomes among treatment groups are reported in Table 4.

#### 3.4.2. Length of Hospital Stay

Concurrent drug treatments, age, EDRC, BMI at admission, DUI, BMI improvement were not significantly correlated with LOS. Thus, potential confounding factors were not included in further analyses. The ANCOVA on LOS revealed a significant difference between treatment groups (F (4, 75) = 5.993, *p* < 0.001). A Bonferroni post-hoc test revealed that the LOS was statistically significantly lower for the early AAP-early NGT group (81.3 ± 31.4 days) compared to late AAP-early NGT (182.6 ± 110.9 days, *p* < 0.001) and late AAP-late NGT (155.4 ± 56.4 days, *p* = 0.026) groups.

## 4. Discussion

At present, the proper timing for the use of specific interventions in the hospital treatment of AN is still the subject of debate. This study is the first one to explore the outcome differences between interventions with both AAP and NGT administered with different timings in the treatment of AN. We divided our population into five groups, according to the introduction of AAP and NGT treatments. A series of relevant differences emerged among treatment groups.

With regard to the length of hospitalization, we found significantly shorter periods for the early AAP-early NGT group (81.8 ± 31.4 days), when compared to the two groups with a later AAP introduction, namely late AAP-early NGT (182.6 ± 110.9 days) and late AAP-late NGT (155.4 ± 56.4 days). No significant difference in the administration of different antipsychotics emerged concerning treatment outcomes. Notably, olanzapine represents the most studied AAP administered to young patients with AN, and a series of studies documents its positive effect on body weight in malnourished patients [32]. In light of these studies, our findings suggest that early AAP introduction may be a contributing factor to shortening the acute hospitalization of AN patients independently of the AAP specifically administered. Since a better outcome was evident only for the early AAP-early NGT group, but not for the “early AAP-late NGT” group, we hypothesize a combined effect of the two early interventions.

Our results appear to be consistent with previous research encouraging the early use of AAP in individuals with schizophrenia spectrum and other psychotic disorders [23,24]. To our knowledge, ours is the first study to document a positive effect of early treatment with AAP in patients with AN. Based on our data, we hypothesize that early use of AAP in selected patients hospitalized for AN may shorten the treatment process of inpatients suffering from acute AN, enabling a quicker transition to a less intensive level of care as recommended by recent guidelines [7].

There is no doubt that AN and schizophrenic patients present clinical differences that make their comparison difficult. However, relevant comorbidities in AN have been documented, with psychotic symptoms occurring in several AN patients [33] and causing poorer outcomes [34]; patients with AN and concurrent psychotic symptoms may be more responsive to medications targeting dopaminergic symptoms, such as AAPs [35,36]. AAPs may influence the outcome of patients with AN with a series of direct and indirect effects, including improvement of compliance, agitation, rigidity, and body image distortion [33]. In our sample, psychopathology was studied using EDI-3 EDRC to measure AN specific symptoms, such as body dissatisfaction, bulimia, and drive for thinness. We did not administer any scale for the specific assessment of psychotic symptoms. Future studies should investigate specific psychopathologic variables, i.e., psychotic symptoms, influencing the response of AN patients to AAPs. Nonetheless, clinicians should keep in mind that medications should never be the first line choice in the treatment of AN [12]. Since evidence for the use of psychopharmacologic treatments in children and adolescents with eating disorders is particularly limited, these interventions should be adopted only in selected patients and treatment settings [7,37].

In our study, a treatment early start was not linked to a greater BMI improvement. Previous research investigating weight gain as an outcome for patients with AN treated with AAP have shown conflicting evidence, with an RCT and a case-control study showing no weight gain associated with treatment with olanzapine [38,39,40]. Other studies have documented partial weight restoration [16]. We hypothesize that the early introduction of AAP does not affect weight difference at discharge directly. It is possible that specific subgroups of patients may show greater benefits.

We did not find significant differences in treatment outcomes for the early use of NGT. Previous studies have documented that a faster restoration of nutritional status may help prevent metabolic complications and reduce anxiety and depressive symptoms [41,42]. Most likely, weight difference at discharge and length of hospital stay are not directly influenced by the earlier or later use of NGT in our sample. It is possible that other outcome measures, such as treatment compliance and nutritional measures, may benefit from early interventions with NGT.

Relevantly, despite differences emerged between groups treated with both AAP and NGT, no difference was found in any of the treatment outcomes between these groups and the control group (NGT only). This could be attributed to differences in the psychopathology presented in patients with AN, with possible effects on the clinical response to both AAP and NGT. AAP, moreover, are sometimes used to address psychopathological factors interfering with compliance to an NGT treatment. Given the presence of these multiple potential confounding factors, we think specific studies should be conducted to assess the efficacy of treatments with NGT only in patients with severe AN.

Our sample included seventy-eight females and only one male. Thus, contributions of differences between genders to clinical outcomes cannot be inferred from this study. A recent systematic review has documented no firm evidence for gender differences concerning outcome and mortality in both adolescents and adults [43]. Hence, insufficient knowledge is available so far to predict specific gender contributions in the prognosis of AN.

In this study, BMI at admission resulted as negatively associated with BMI improvement. Thus, patients with lower starting BMI were found to have a better weight outcome, independently from age and received treatments. This apparently contradictory finding may be considered in the light of a recent study, documenting that total weight loss and recent weight loss were better predictors than admission weight of many physical complications [44]. Hence, we may hypothesize that a series of anamnestic clinical data, apart from admission BMI, should be consider when trying to properly assess the severity of patients with AN admitted to hospital services.

Our study presents a few limitations. An untreated comparison group was not included, since we intended to investigate the specific timing of treatment options in severely metabolically impaired patients. The retrospective nature and the lack of follow-up data may represent further limitations, which need to be addressed by future studies.

Moreover, all the patients included in this sample received a specific third-level inpatient treatment regime, characterized by psychiatric, nutritional and psychological assessments and interventions. Thus, the results and conclusions of this study should be cautiously generalized to clinical populations treated in different settings, i.e., pediatric or psychiatric departments.

Nevertheless, this study also presents remarkable characteristics and data: (a) it addresses a homogeneous sample of adolescents affected by AN; (b) it is the first study to address the lack of evidence on the timing of pharmacological and nutritional interventions occurring concurrently; (c) different from most research published on pharmacological treatment, it collects and review data on the use of four different antipsychotic drugs (olanzapine, risperidone, aripiprazole, and quetiapine).

This study provides new interesting and helpful data to stakeholders in the field of ED. It is important for the clinicians, for the patients and their relatives to acknowledge that the timing of interventions with AAP and/or NGT in the inpatient treatment of AN may impact the final outcomes. Public health professionals, moreover, should investigate the possibility that the length of hospital stay of inpatients with AN could be influenced by the timing of the administered interventions. Future research in the field of AN should assess the validity of these results in greater samples and acknowledge the timing of AAP and NGT interventions as a potentially relevant variable influencing the outcomes in clinical studies.

## 5. Conclusions

Our study documents a reduced length of hospital stay for patients treated with early AAP and early NGT interventions. We consider this result to be relevant for clinical practice, and deserving of further examination, for example in case-control studies with larger samples of inpatients.

## Figures and Tables

**Table 1:** Demographic characteristics of the sample. Continuous variables expressed as mean (±SD).

Variables	Early AAP-Early NGT (EE)	Early AAP-Late NGT (EL)	Late AAP-Early NGT (LE)	Late AAP-Late NGT (LL)	NGT Only (NGT)	*p*-Value
Number of patients	18	12	20	18	11	/
Gender	F = 18 (100%); M = 0 (0%)	F = 11 (91.7%); M = 1 (8.3%)	F = 20 (100%); M = 0 (0%)	F = 18 (100%); M = 0 (0%)	F = 11 (100%); M = 0 (0%)	0.220
Age at admission (years)	14.7 (±2.3)	14.5 (±1.8)	14.3 (±1.7)	15.2 (±1.5)	14.2 (±2.0)	0.606
Diagnosis	ANR = 17 (94.4%); ANBP = 1 (5.6%)	ANR = 12 (100%); ANBP = 0 (0%)	ANR = 20 (100%); ANBP = 0 (0%)	ANR = 18 (100%); ANBP = 0 (0%)	ANR = 11 (100%); ANBP = 0 (0%)	0.480
PD	1 (5.6%)	0 (0%)	0 (0%)	0 (0%)	0 (0%)	0.363
MDD	1 (5.6%)	0 (0%)	0 (0%)	1 (5.6%)	1 (9.1%)	0.679
OCD	0 (0%)	1 (5.6%)	1 (5.6%)	4 (22.2%)	0 (0%)	0.083
Duration of illness before admission (months)	15.8 (±14.3)	10.3 (±6.3)	12.7 (±9.3)	19.0 (±13.0)	15.0 (±10.3)	0.297
SSRI	16 (88.9%)	12 (100%)	20 (100%)	17 (94.4%)	10 (90.9%)	0.328
EDI-3 EDRC at admission	73.5 (±20.2)	65.6 (±26.6)	66.7 (±21.1)	82.5 (±12.0)	80.0 (±13.0)	0.252
BMI at admission (kg/m^2^)	14.2 (±1.9)	13.5 (±1.6)	13.3 (±1.6)	13.6 (±1.2)	13.8 (±1.6)	0.360
BMI difference (kg/m^2^)	1.6 (±1.2)	2.3 (±1.4)	2.6 (±1.4)	1.4 (±1.1)	1.8 (±1.2)	**0.027**
LOS (days)	81.8 (±31.4)	114.7 (±72.4)	182.6 (±110.9)	155.4 (±56.4)	109.6 (±32.5)	**<0.001**

Abbreviations: EE: early AAP-early NGT; EL: early AAP-late NGT; LE: late AAP-early NGT; LL: late AAP-late NGT; ANR: anorexia nervosa, restrictive subtype; ANBP: anorexia nervosa, binge-purging subtype; BMI: body-mass index; EDI-3: Eating Disorders Inventory-3; EDRC: eating disorders risk; MDD: major depressive disorder; SSRI: selective serotonin reuptake inhibitors; LOS: length of in-hospital stay; OCD: obsessive-compulsive disorder; PD: panic disorder. Note: statistically significant differences marked in bold and underlined.

**Table 2:** Correlations between outcome variables and demographic, clinical, treatment and outcome variables.

Outcome Variables		
Variables	BMI improvement	LOS
**Demographic variables**		
Sex	*p* = 1.000	*p* = 1.000
Age at admission	rho = −0.416, ***p *< 0.001**	rho = 0.087, *p* = 0.445
**Clinical variables at admission**		
DUI	rho = −0.248, *p* = 0.027	rho = 0.162, *p* = 0.155
Admission BMI	rho = −0.401, ***p *< 0.001**	rho = −0.179, *p* = 0.122
Admission EDRC	rho = −0.174, *p* = 0.265	rho = −9.8^−4^, *p* = 0.995
Diagnosis	*p* = 1.000	*p* = 1.000
OCD	*p* = 0.262	*p* = 0.044
PD	*p* = 1.000	*p* = 1.000
MDD	*p* = 0.741	*p* = 0.382
**Treatment variables**		
Early AAP	*p* = 0.622	*p* < 0.001
Early NGT	*p* = 0.365	*p* = 0.161
SSRI	*p* = 0.037	*p* = 0.011
Fluvoxamine	*p* = 0.260	*p* = 0.289
Sertraline	*p* = 0.415	*p* = 0.252
Fluoxetine	*p* = 0.710	*p* = 0.155
**Outcome variables**		
BMI improvement	/	rho = 0.200, *p* = 0.075
LOS	rho = 0.200, *p* = 0.075	/

Abbreviations: BMI: body-mass index; EDI-3: Eating Disorders Inventory-3; EDRC: eating disorders risk; MDD: major depressive disorder; SSRI: selective serotonin reuptake inhibitors; LOS: length of in-hospital stay; OCD: obsessive-compulsive disorder; PD: panic disorder. Note: Bonferroni corrected significance values: demographic variables: 0.05/2 = 0.025; clinical variables: 0.05/7 = 0.007; treatment variables: 0.05/6 = 0.008; outcomes variables: 0.05/2 = 0.025. Statistically significant results marked in bold and underlined.

**Table 3:** Treatments with different AAPs in the whole sample. Continuous variables expressed as mean (±SD).

Variables	Olanzapine	Aripiprazole	Risperidone	Quetiapine
N. of patients treated	32 (47.1%)	25 (36.7%)	14 (20.6%)	8 (11.8%)
Mean maximum dosage (in mg)	6.1 (±2.3)	10.4 (±3.4)	0.7 (±0.3)	125.0 (±97.3)
Admission-discharge BMI difference (in kg/m^2^)	2.2 (±1.3)	1.7 (±1.5)	2.7 (±1.2)	1.2 (±1.2)
*p*-value of differences in Admission-discharge BMI difference	0.205	0.279	0.033	0.112
LOS (in days)	138.6 (±81.9)	146.9 (±71.3)	96.0 (±30.0)	165.5 (±153.6)
*p*-value of differences in LOS (in days)	0.902	0.083	0.022	0.917

Abbreviations: BMI: body-mass index; LOS: length of in-hospital stay. Note: Bonferroni-corrected significance level = 0.05/4 = 0.0125.

**Table 4:** Comparisons of treatment outcomes among treatment groups.

	EE	EL	LE	LL	NGT	
EE	n/a	1	**<0.001**	**0.025**	1	LOS (*p*-value)
	n/a	1	0.894	1	1	BMI (*p*-value)
EL	/	n/a	0.107	1	1	LOS (*p*-value)
	/	n/a	1	0.92	1	BMI (*p*-value)
LE	/	/	n/a	1	0.077	LOS (*p*-value)
	/	/	n/a	0.154	1	BMI (*p*-value)
LL	/	/	/	n/a	0.509	LOS (*p*-value)
	/	/	/	n/a	0.966	BMI (*p*-value)
NGT	/	/	/	/	n/a	LOS (*p*-value)
	/	/	/	/	n/a	BMI (*p*-value)

Abbreviations: NGT: naso-gastric tube feeding; EE: early AAP-early NGT; EL: early AAP-late NGT; LE: late AAP-early NGT; LL: late AAP-late NGT; LOS: length of in-hospital stay. Note: each cell reports *p*-values of comparisons on both outcomes: LOS (upper value) and BMI difference (lower value). Statistically significant differences marked in bold and underlined.

## Data Availability

The datasets used and analyzed during the current study are available from the corresponding author on reasonable request.
